# Do general practice patients with and without appointment differ? Cross-sectional study

**DOI:** 10.1186/s12875-018-0787-5

**Published:** 2018-06-23

**Authors:** Bernhard Riedl, Simon Kehrer, Christoph U. Werner, Antonius Schneider, Klaus Linde

**Affiliations:** Technical University of Munich, TUM School of Medicine, Institute of General Practice, Orleansstrasse 47, 81667 Munich, Germany

**Keywords:** Primary care, Walk-in patients, Organization, Waiting times, Mental disorders

## Abstract

**Background:**

Even in practices with a comprehensive appointment system a minority of patients walks in without prior notice, sometimes causing problems for practice service quality. We aimed to explore differences between patients consulting primary care practices with and without appointment.

**Methods:**

Consecutive patients visiting five primary care practices without an appointment and following patients with an appointment were asked to fill in a four-page questionnaire addressing socio-demographic characteristics, the reason for encounter, urgency of seeing a physician, depressive, somatic and anxiety symptoms, personality traits, and satisfaction with the practice. Physicians also documented the reason for encounter and assessed the urgency. Data were analyzed using univariate and multivariate methods.

**Results:**

Two hundred fifty-one patients without and 250 patients with appointment participated. Patients without appointment were significantly younger (mean age 44 vs. 50 years) and reported less often chronic diseases (29% vs. 45%). Also, reasons for encounter differed (e.g., 27% vs. 16% with a respiratory problem). Patients’ ratings of urgency did not differ between groups (*p* = 0.46), but physicians rated urgency higher among patients without appointment (*p* < 0.001). In logistic regression analyses younger age, male gender, absence of chronic disease, positive screening for at least one mental disorder, low values on the personality trait openness for experience, a high urgency rating by the physician, and a respiratory or musculoskeletal problem as reason for encounter were significantly associated with a higher likelihood of being a patient without appointment.

**Conclusions:**

In this study, younger age and a high urgency rating by physicians were the variables most consistently associated with the likelihood of being a patient without appointment. Overall, differences between patients seeking general practices with a comprehensive appointment system without prior notice and patients with appointments were relatively minor.

## Background

Service-time planning in primary care is a complex issue. Specific challenges arise both on the level of interday (over more than one day) and intraday (within a single day) scheduling [1]. A specific challenge are urgent (who really need care on the same day) and walk-in (who come in without making an appointment) patients. There are basically three strategies for how practices can handle such problems [2]. In the traditional model the working time of the physician is completely booked in advance. Urgent or walk-in patients often lead to double-booking and cause long waiting times. In the curve-out model the urgent demand is predicted based on previous experience and fixed capacities are reserved for urgent and walk-in patients. The advanced access model tries to give all patients an appointment on the same day rejecting the idea of sorting the demand into routine and urgent. While the advanced access model has been shown to be efficient in primary care clinics in reducing the waiting time for an appointment [3], it is unlikely to solve the problem of increased waiting times at the time of appointment in small practices when additional walk-in patients show up.

In Germany, most general practitioners work in a single-handed practice or a small group practice. We could not identify any empirical studies on scheduling styles. According to the authors’ experience, only a minority of practices use a comprehensive appointment system that requires all patients to call and make an appointment prior to a practice visit. Empirical studies in German practices show that the introduction of such an approach reduced waiting times [4], yet about 5% of patients still come without calling for an appointment before [5, 6]. Most of these patients do not require immediate emergency care.

While a number of studies compare the characteristics of patients of “normal” general practices with those of practices or clinics specialized in walk-in and urgent patients or after-hours services (e.g. [7]), we are not aware of any studies investigating differences between walk-in patients and patients making an appointment in normal general practices. Such studies seem desirable to provide reliable information for a better understanding of reasons for non-emergency practice visits without appointment and to facilitate the development of strategies for reducing unnecessary visits.

A variety of factors might influence why patients visit practices without making appointments. It seems plausible that a worsening of complaints, a high urgency, a pending or immediately preceding weekend increase the likelihood of such behavior. Younger patients might have a stronger consumer attitude than older patients expecting that their physician is always available for them [8]. In discussion with practitioners we sometimes heard that physicians had the impression that “difficult” patients with psychological problems (such as depression, anxiety or somatoform disorders) or specific personality traits (e.g., neuroticism) are over-represented among patients without appointment. This would be in line with the finding that patients with psychological or psychosomatic disorders have increased utilization rates [9, 10]. Finally, it seems obvious to assume that conditions are more often acute than among average practice patients.

In the study reported below we aimed to explore whether patients consulting primary care practices with and without appointment differ with regard to the following variables: a) socio-demographic characteristics; b) reason for encounter; c) the urgency of seeing a physician (both from the patient‘s and the physician’s perspective); d) depressive, somatic and anxiety symptoms; e) personality traits; and f) satisfaction with the practice. In addition, we tested three hypotheses: 1) patients without appointment consider the urgency of seeing the physician higher than patients with appointment; 2) the difference between patients’ and physicians’ perception of urgency is more pronounced among patients without appointment; 3) a positive screening result for mental co-morbidity is more frequent among patients without appointment with low urgency (physician perspective) compared to patients without appointment but high urgency and patients with appointment.

## Methods

### Study design, setting, ethics and patient selection

This was a comparative cross-sectional study with two groups (patients with and without appointment). It was performed in five general practices (one urban, four rural) with a comprehensive appointment system in Bavaria, Germany. All participating practices aim to make appointments with *all* patients (i.e., patients with acute complaints are asked to inform the practice prior to their visit, too; time slots are pre-planned for urgent patients). The study protocol was approved by the ethics committee of the Medical Faculty of the Technical University of Munich (reference number 335/15).

All eligible consecutive patients visiting the participating practices without appointment were informed about the study and invited to participate. Inclusion criteria were an age of at least 18 years, sufficient skills of German language to fill in questionnaires and consent to participation. We excluded patients coming only to the practice for picking up a prescription, who did not aim to see the physician, or who needed immediate emergency care. If a patient without appointment agreed to participate, the next patient with appointment was invited to participate, too. If this patient did not give consent, the next patient with appointment was invited. This approach was chosen to include a representative sample of patients with appointments visiting the practices at the same days and time of day as patients without appointment. In order to avoid selection bias, patients were recruited strictly in the order in which they had actually entered the practice.

### Questionnaire

All participants were asked to fill in an anonymous four-page questionnaire. The first section addressed socio-demographic characteristics and duration of participants’ affiliation to the practice. The second section started with an open question asking participants to report their complaints/reasons for seeing the physician. In addition, patients were asked to go through a list of reasons (taken from a previous survey [5]) why they came to the practice right now. All participants then were asked to rate the urgency of seeing the doctor (on a five-point verbal rating scale from “not urgent at all” to “very urgent”) and to report any chronic diseases. The third section included German versions of three validated instruments to screen for depression, somatoform disorder and generalized anxiety disorder. The widely used Patient Health Questionnaire for Depression (PHQ-9) [11] comprises nine items which score each of the DSM-IV criteria (Diagnostic and Statistical Manual of Mental Disorders). The PHQ-15 [12] comprises 15 items to screen for somatization and to monitor somatic symptom severity. The Generalized Anxiety Disorder Assessment (GAD-7) [13] includes a total of seven items. All three scales can be analysed dimensionally (resulting in a score) and categorically (resulting in a tentative diagnosis). The third section also included a new scale, the Somatic Symptom Disorder - B Criteria Scale (SSD-12) which was developed as a direct measure based on the re-conceptualized psychological criteria of Somatic Symptom Disorder [14]. The results of the psychometric evaluation of this instrument in our sample of primary care patients are reported elsewhere [15]. In the fourth section of the questionnaire patients were asked to fill in a German 21-item version of the Big Five Inventory (BFI-K) to investigate personality traits [16]. The Big-Five is a widely examined theory of five broad dimensions to describe the human personality. The five factors are extraversion, neuroticism, openness to experience, conscientiousness, and agreeableness. In the last section participants were asked to rate their satisfaction with waiting times, organization, the physician, non-medical staff and with the practice in general using German school grades (from 1 = very good to 6 = unsatisfactory). Physicians only documented weekday and month, the reason for encounter as reported by the patient, the result of the encounter as free text and the same rating of urgency as patients. For the evaluation, all free text information was coded according to ICPC-2 (International Classification of Primary Care, version 2 [17]). The doctors did not see the completed questionnaires of the patients. Names of patients were not documented anywhere in the study documentation; this also implies that consent was exclusively oral.

### Statistics

All statistical analyses were performed using SPSS 23. Findings were summarized descriptively as absolute frequencies (percentages), medians (ranges) or means (standard deviations) depending on scale level and distribution. Differences between groups were investigated in an explorative manner using Chi^2^-tests, Mann-Whitney U-tests or Student’s t-tests. We did not adjust *p*-values for multiple testing. Ninety-five percent confidence intervals for the proportion of patients recruited per weekday were estimated with bootstrapping. We used the intra-class correlation coefficient (ICC; two-way mixed model) to assess the agreement of urgency ratings from patients and physicians. To further explore which factors might be independently associated with the likelihood of being a patient without appointment we performed multivariate logistic regression analyses. Selection of variables was based on explicit study aims (see introduction), group differences identified in univariate analyses, correlational analyses, and completeness of data. To control for potential center effects we included practices as dummy variables in the calculations. We used both inclusion and (backwards and forwards) selection models; findings were consistent. To limit the number of variables we present the Wald backwards model selection in the results section. When planning the study we performed a sample calculation using G*Power 3.1.9. For detecting a standardized mean difference of 0.3 between the two groups regarding urgency rating with a power of 90% (α = 5%) a sample size of 470 (2 × 235) patients was needed. The recruitment target was set at 500 patients to account for missing data.

## Results

Between October 2015 and April 2016, a total of 501 patients were included in the study. About 50 patients approached (similarly distributed among patients with and without appointment) refused to participate, typically because of lack of time or unwillingness to fill in the questionnaire. Among the 501 participants, patients without appointment were younger than those with appointments (mean 44 vs. 50 years), reported less often chronic diseases (29% vs. 45%; independently from the current reason for encounter), were more often living without a partner or unmarried in partnership, less often had children, and tended to be better educated (see Table 1). Significantly more patients were recruited on a Monday (27%); recruitment on the remaining weekdays varied within the limits expected by chance.

**Table 1:** Characteristics of participants

Variable (missing values)	Patients without appointment (*n* = 251)	Patients with appointment (*n* = 250)	*p*-values for group differences
Age in years (4)	44 (16)	50 (16)	< 0.001§
Female (3)	120 (48%)	139 (56%)	0.11*
Family status (4)			
- single	77 (31%)	56 (23%)	
- unmarried in partnership	38 (15%)	21 (8%)	
- married	129 (51%)	155 (63%)	0.005*
- widowed	7 (3%)	14 (6%)	
At least one child (3)	154 (61%)	178 (72%)	0.01*
Privately insured (5)	22 (9%)	21 (13%)	0.19*
High school graduation (10)	82 (33%)	63 (24%)	0.07*
Patient in the practice since … years (60)	8 (2, 15)	8 (4, 20)	0.13#
At least one chronic disease (3)	71 (29%)	113 (45%)	< 0.001

Values are means (standard deviations) for age, median (1. and 3. quartile) for years in practice, and absolute frequencies (percentages) for other variables. § = *p*-values from Student‘s t-test; # = from Mann-Whitney-U-Test; * = from chi^2^-test

**Fig. 1:**
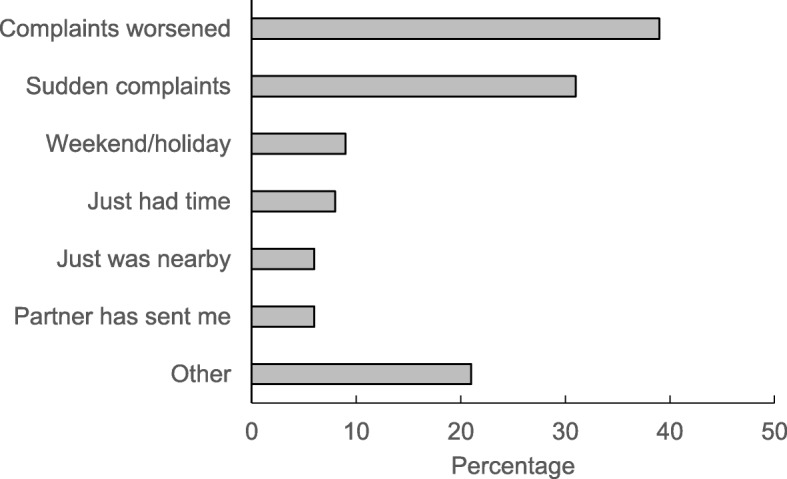
Reasons for visiting the practice among patients without appointment (*n* = 251)

Among patients without appointment the most frequent reason of the consultation classified as ICPC-2 category was a respiratory problem (27%), followed by musculoskeletal problems (25%). Among patients with appointment procedures were the most frequent reason (24%), followed by musculoskeletal (18%) and respiratory problems (16%). Table 2 also gives an overview of the most frequent single ICPC-2 codes. The most frequent motive for visiting the practice just now without appointment were worsening complaints (39%) and a weekend or holiday approaching or preceding (31%), but 14% ticked the answer options “I just had time” or “I just was nearby” (Fig. 1).

**Table 2:** Categories and codes for reasons for encounter (as reported by physicians) according to the International Classification of Primary Care, version 2 (ICPC-2)

	Patients without appointment (*n* = 251)	Patients with appointment (*n* = 250)	*P* values for group differences
ICPC-2 categories			
Respiratory (R)	68 (27%)	41 (16%)	0.005
Musculoskeletal (L)	62 (25%)	45 (18%)	0.08
Process codes (−)	17 (7%)	60 (24%)	< 0.001
Digestive (D)	27 (11%)	12 (5%)	0.02
Cardiovascular (K)	13 (5%)	16 (6%)	0.57
General and unspecified (A)	15 (6%)	13 (5%)	0.14
Psychological (P)	15 (6%)	12 (5%)	0.69
Other	35 (13%)	41 (16%)	0.59
Most frequent single ICPC-2 codes			
Upper respiratory infection acute (R74)	35 (14%)	22 (9%)	0.07
Back syndrome w/o radiating pain (L84)	15 (6%)	8 (3%)	0.14
Blood test (−34)	1 (< 1%)	18 (7%)	nc
Gastroenteritis (D73)	14 (6%)	3 (1%)	nc
Preventive immunization/medication (−44)	4 (2%)	11 (4%)	nc
Therapeutic counsellling/listening (−58)	8 (3%)	7 (3%)	nc
Neck syndrome (L83)	10 (4%	4 (2%)	nc
General symptom/complaint other (A29)	9 (4%)	3 (1%)	nc
Sinusitis acute/chronic (R75)	6 (2%)	4 (2%)	nc
Acute bronchitis/bronchiolitis (R78)	5 (2%)	4 (2%)	nc
Bursitis/tendinitis/synovitis (L87)	2 (1%)	6 (2%)	nc

Values are absolute frequencies (percentages). *P*-values from Fisher’s exact test; nc = not calculated (as total frequency in all participants < 20)

While in general patients rated the urgency of seeing the doctor higher than physicians, contrary to our hypothesis 1 patients without appointment did not consider the reasons for their visit more urgent than patients with appointment (Table 3). However, 66 of 250 patients with appointment (compared to only seven patients without appointment) did not answer the question on urgency. Almost half (32) of these patients had come for a procedure (compared to only two among patients without appointment). Physicians considered urgency significantly higher in patients without appointment than in patients with appointment (28% very urgent or urgent compared to 16%). Among participants with ratings both from patients and physicians, patients with appointment rated the urgency of their visit more often higher than physicians compared to patients without appointment (contradicting our hypothesis 2). Agreement between patient and physician ratings of urgency was poor to fair (ICC = 0.33; 95% CI 0.11 to 0.48).

**Table 3:** Assessment of urgency by patients and doctors and differences between the two

Rating (Code)	Patients without appointment (*n* = 251)	Patients with appointment (*n* = 250)	*p*-value
Patient’s view			
Very urgent	24 (10%)	15 (8%)	
Urgent	100 (41%)	66 (36%)	
Somewhat urgent	81 (3%)	79 (43%)	0.46
Not urgent	35 (14%)	23 (12%)	
Not urgent at all	5 (2%)	1 (1%)	
Missing	7	66	
Physician‘s view			
Very urgent	15 (6%)	8 (3%)	
Urgent	56 (22%)	33 (13%)	
Somewhat urgent	84 (34%)	60 (24%)	< 0.001
Not urgent	57 (23%)	91 (37%)	
Not urgent at all	37 (15%)	57 (23%)	
Missing	2	1	
Difference physician – patient			
Patient very much more urgent (−4)	3 (1%)	1 (< 1%)	
Patient much more urgent (−3)	15 (6%)	16 (8%)	
Patient more urgent (−2)	33 (14%)	43 (23%)	
Patient slightly more urgent (−1)	72 (30%)	53 (29%)	0.007
Full agreement (0)	76 (31%)	49 (29%)	
Physician slightly more urgent (1)	36 (15%)	17 (9%)	
Physician more urgent (2)	8 (3%)	5 (3%)	
Physician much more urgent (3)	–	–	
Physician very much more urgent (4)	–	–	
Missing	8	66	

Values are absolute frequencies (percentages). *P*-values from Mann-Whitney U-test*

Mean scores for symptoms of depression, somatoform symptoms, psychological criteria of somatic symptoms disorder and generalized disorder did not differ significantly between participants with and without appointments (Table 4). Also, there were no differences between the two groups regarding the frequency of the tentative diagnoses major depression, somatoform disorder and generalized anxiety disorder. However, 13% of patients without appointment fulfilled the criteria of minor depression compared to 4% of patients with appointment, and in 28% compared to 21% screened positive for at least one mental disorder. The frequency of at least one suspected mental disorder among patients without appointment and a low urgency rating by the physician (28%) did not differ significantly from that among patients with a higher urgency rating (some urgency 25%, higher urgency 34%; *p* = 0.47 across all three groups) and patients with appointment (21%; *p* = 0.19).

**Table 4:** Results of screening for depression, somatoform disorder or generalized anxiety disorder

Symptoms/disorder - instrument (n missing)	Patients without appointment (*n* = 251)	Patients with appointment (*n* = 250)	*p*-value
Metric analysis (scores)			
Depressive symptoms - PHQ-9 (34)	5.2 (4.9)	5.0 (4.8)	0.64
Somatoform symptoms - PHQ-15 (34)	6.8 (5.2)	6.8 (4.8)	0.96
Somatic symptom disorder – SSD-12 (39)	11.6 (8.7)	11.3 (9.3)	0.43
Generalized anxiety symptoms - GAD-7 (70)	4.1 (4.3)	3.7 (3.9)	0.32
Categorical analysis			
Major depression - PHQ-9 (34)	19 (8%)	17 (7%)	0.731
Minor depression - PHQ-9 (34)	30 (13%)	9 (4%)	< 0.001
Somatoform disorder - PHQ-15 (70)	34 (16%)	34 (16%)	1.00
Generalized anxiety disorder - GAD-7 (34)	24 (10%)	21 (9%)	0.64
At least one mental disorder suspected*	71 (28%)	52 (21%)	0.06

*P*-values from Student’s t-test or Chi^2^-test; *missings were coded as no indication of a mental disorder

Values are means (standard deviations) or absolute frequencies (percentages)

There were no significant group differences regarding personality traits and satisfaction with the practice.

In logistic regression analyses (see Table 5) younger age, male gender, absence of chronic disease, positive screening for at least one mental disorder, low values on the personality trait openness for experience, a high urgency rating by the physician, and an ICPC-2 category respiratory or musculoskeletal problems as reason for encounter were significantly associated with a higher likelihood of being a patient without appointment. The ICPC-2 category process code was less frequent among patients without appointment. Urgency as perceived by patients was not included in the final regression analyses due to the high number of missing values. In sensitivity analyses including this variable, it was not associated with group status (*p* > 0.2).

**Table 5:** Logistic regression analysis investigating which factors are associated with a higher likelihood of being a patient without appointment (Wald backwards selection, r^2^ = 0.25; *n* = 454)

Variable	β	*p*-value	OR (95%-CI)
Age (per year)	−0.03	< 0.001	0.97 (0.96; 0.99)
Male gender	0.56	0.01	1.75 (1.14; 2.69)
At least one chronic disease	−0.55	0.02	0.58 (0.36; 0.91)
Screened positive for at least one mental disorder	0.55	0.03	1.73 (1.07; 2.79)
Openness for experience	−0.31	0.04	0,73 (0.54; 0.99)
Urgency (physician rating)	−0.38	< 0.001	0.68 (0.56; 083)
ICPC category respiratory	0.63	0.03	1.89 (1.08; 3.30)
ICPC category digestive	0.82	0.06	2.72 (0.98; 5.28)
ICPC category musculoskeletal	0.57	0.04	1.77 (1.02; 3.05)
ICPC category process codes	−0.83	0.03	0.44 (0.21; 0.91)

*β* regression coefficient, *OR* odds ratio, *95%-CI* 95% confidence interval. Variables excluded in the selection process: education, family status, children, insurance status, extraversion, neuroticism, conscientiousness, and agreeableness. Analyses controlled for center effects

## Discussion

### Summary of main findings

In this exploratory study patients consulting practices with a comprehensive appointment system tended to be younger, more often male, less often chronically ill and had somewhat different reasons for encounter if appearing without an appointment. Contrary to our expectations these patients did not rate the urgency of seeing a doctor higher than patients with appointment while physicians actually did (suggesting that patients without appointment more often have more urgent problems). Mental morbidity might indeed be higher among patients without appointment, however, our findings were not fully consistent across analyses.

### Strengths and limitations

As far as we know this is the first study systematically investigating differences between walk-in patients and patients making an appointment in general practices. The comparative cross-sectional study design we chose to answer our study questions is somewhat unusual. Our strategy of consecutively including patients without appointment and the very next following patients with appointment has the advantage to make sure that days and time of recruitment are well comparable in both groups. When planning the study we had considered the alternative of taking a random sample of *all* patients with appointment as control group. This would have meant that participants would have been representative for practice patients *in general* (however, ignoring that weekday and time of the day might have an influence). As this approach would have been much more difficult to implement we opted for the first approach. A methodological weakness of our study is that the documentation of eligible patients refusing participation was not implemented as planned in the protocol (probably because we failed to emphasize the importance of this issue to the practice staff). Age, gender and reasons for refusal were not documented consistently and from interviews with the staff members we know that sometimes it was forgotten to register a non-responder. While we are certain that the number of non-responders was around 50 and similarly distributed among both groups we cannot say whether age and gender of participants and non-responders differed. Yet, given the low non-response-rate of about 10% it is very unlikely that study participants are a strongly biased sample. Reasons for encounter of participants among patients with appointment were very similar to those typically seen in German primary care practices [18]. Obviously, our findings have to be interpreted within the framework of the German primary health care system with mostly small general practices. Only 5 practices participated in our study and we can not be sure that they were representative, even though they were typical GP practices. As we did not adjust for multiple testing the significant differences between groups from univariate analyses must be interpreted with caution. The multivariate logistic regression analysis provides more reliable evidence on which factors influence the likelihood of seeking a practice without appointment. Yet, also these findings have to be considered exploratory and need independent replication.

### Interpretation

A number of studies have shown that waiting times in primary care strongly influence patient satisfaction. Longer waiting times do not only lead to dissatisfaction of patients with organization but can induce concerns about the medical quality of practices and physicians [19, 20]. Although shorter waiting times have been shown for practices with a structured appointment system [4], a number of patients still appear in these practices without appointment even if there is no need for immediate emergency treatment.

Yet, our finding that physicians, on average, rate urgency higher among patients without appointments suggests that their behavior might be justified to some extent in a number of cases. Studies in emergency departments show that, on average, frequent users tend to be sicker than occasional users although many emergency visits might not be justified [21]. To our surprise urgency ratings of patients did not differ between groups and there was no association of this variable with the likelihood of being a patient without appointment in multivariate regression analyses. The high number of missing answers for this item among patients with appointment (particularly, among patients coming for a procedure only) might explain why a difference was not observed in the univariate analysis. But this explanation applies less to the multivariate analyses which adjust for differences between groups regarding reasons for encounter, age and other variables. In any case, our results suggest that self-perceived urgency of symptoms is not necessarily the main reason for a patient to visit a practice without an appointment. Low subjective urgency ratings are also frequent among patients seeking care in emergency departments [22].

Beside a high urgency rating of the physician younger age was the factor most consistently associated with the likelihood of being a patient without appointment. This finding fits well with what we expected and what is known in related settings. Studies investigating the crowding of outpatient emergency departments also found that patients with a relatively young age are over-represented [21], particularly among patients with low subjectively perceived treatment urgency [22]. A variety of reasons, such as time constraints due to employment and young children as well as a stronger consumer attitude [8] might explain such an association. The group differences regarding family status, having children or not, and education in our univariate analyses are at least partly explained by age differences. In international studies, frequent use of emergency departments is often associated with female sex [21]. On the opposite, several studies among walk-in patients in out-of-hours GP services or emergency departments found that young males were over-represented [22–25]. In our regression analyses we found a significant association of male gender and the likelihood of being a patient without appointment. One possible explanation could be that in the rural setting of our study, men were more likely to work full-time than women. However, as we did not document employment status and working hours per week we were unable to investigate this empirically.

The differences between groups we found regarding reasons for encounter and whether patients (also) suffered from a chronic disease confirm the assumption that patients seeking a practice without appointment more often suffer from acute complaints. This finding again fits well with most studies on frequent users of emergency departments [21].

One motive for carrying out our study was the subjective perception of the participating physicians that “difficult patients” are over-represented among patients without appointments. Our standardized psychometric instruments might not be the most efficient tools to identify patients considered as “difficult” by physicians. However, based on existing evidence showing a link between resource use and mental disorders [9, 10, 26], conceptual and feasibility considerations we considered the use of widely used and validated instruments for screening for mental disorders and for measuring personality treats as straightforward. Our findings are not easy to interpret. While a positive screening result for at least one mental disorder was significantly associated with the likelihood of being a patient without appointment, we neither found group differences for depression, anxiety and somatization scores in univariate analyses nor an association when entering the scores into the regression model instead of the dichotomous summary variable. We cannot rule out that the association found in our main analysis is a chance finding. The same is true for the negative association with the personality trait openness for experience. Even if the identified associations are real, they do not seem to be very strong. Maybe practitioners overestimate the proportion of “difficult” patients in patients without appointment. This biased perception could be due to the additional expense these patients mean to practice organization, working times and individual stress levels [27]. We made a similar experience in a study among patients who request referral without prior assessment [26]. Our data suggest that social and cultural factors might be more relevant for consulting the practice without prior notice. This finding is similar to that found for the unjustified use of emergency departments [22, 25, 28].

## Conclusion

Our study results indicate that patients who visit family practices with a comprehensive appointment system without prior notice differ only slightly from patients with appointment. Younger age seems to be a relevant factor. Qualitative studies could be an efficient way to further investigate why patients with non-urgent complaints come to practices without an appointment. Such studies could help to plan future quantitative studies and to facilitate strategies to approach this problem. Future quantitative studies might focus more on patients without appointment considered as “difficult” by physicians.

## Abbreviations

95% CI: 95% confidence interval
BFI-K: Big Five Inventory short version
GAD-7: Generalized Anxiety Disorder 7-item scale
ICC: intra-class correlation coefficient
ICPC-2: International Classification of Primary Care, version 2
nc: not calculated
PHQ-15: Patient Health Questionnaire 15-Item Somatic Symptom Severity Scale
PHQ-9: Patient Health Questionnaire for Depression
SSD-12: Somatic Symptom Disorder – B Criteria Scale
